# TRBP–Dicer interaction may enhance HIV-1 TAR RNA translation via TAR RNA processing, repressing host-cell apoptosis

**DOI:** 10.1242/bio.050435

**Published:** 2020-02-25

**Authors:** Chiaki Komori, Tomoko Takahashi, Yuko Nakano, Kumiko Ui-Tei

**Affiliations:** 1 Department of Biological Sciences, Graduate School of Science, The University of Tokyo, Hongo, Tokyo 113-0033, Japan; 2 Department of Computational Biology and Medical Sciences, Graduate School of Frontier Sciences, The University of Tokyo, Kashiwano-ha, Chiba 277-8561, Japan

**Keywords:** TRBP, Dicer, TAR miRNA, Infection, Translational regulation

## Abstract

The transactivating response (TAR) RNA-binding protein (TRBP) has been identified as a double-stranded RNA (dsRNA)-binding protein, which associates with a stem-loop region known as the TAR element in human immunodeficiency virus-1 (HIV-1). However, TRBP is also known to be an enhancer of RNA silencing, interacting with Dicer, an enzyme that belongs to the RNase III family. Dicer cleaves long dsRNA into small dsRNA fragments called small interfering RNA or microRNA (miRNA) to mediate RNA silencing. During HIV-1 infection, TAR RNA-mediated translation is suppressed by the secondary structure of 5′UTR TAR RNA. However, TRBP binding to TAR RNA relieves its inhibitory action of translation and Dicer processes HIV-1 TAR RNA to generate TAR miRNA. However, whether the interaction between TRBP and Dicer is necessary for TAR RNA translation or TAR miRNA processing remains unclear. In this study, we constructed TRBP mutants that were unable to interact with Dicer by introducing mutations into amino acid residues necessary for the interaction. Furthermore, we established cell lines expressing such TRBP mutants. Then, we revealed that the TRBP–Dicer interaction is essential for both the TAR-containing RNA translation and the TAR miRNA processing in HIV-1.

## Abstract (precis)

**Summary:** HIV-1 RNA translation may be enhanced through TAR RNA processing by TRBP in collaboration with Dicer, repressing host–cell apoptosis.

## INTRODUCTION

**Fig. 1.**
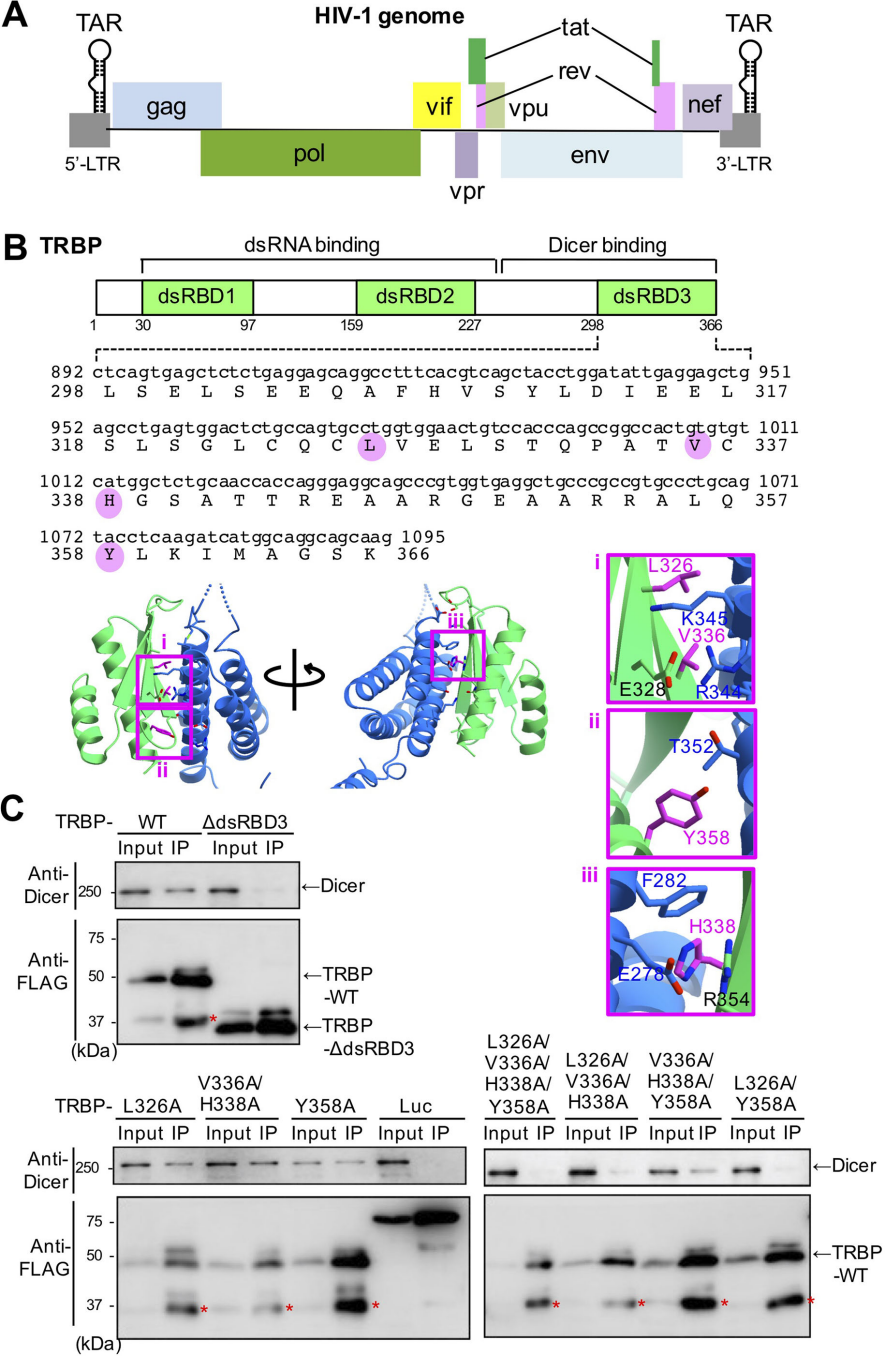
**Construction of mutant TRBP lacking the interaction with Dicer.** (A) Domain structure of the HIV-1 genome. HIV-1 has TAR regions at the 5′- and 3′-LTRs. The HIV-1 genome has genes encoding gag, pol, vif, vpr, rev, tat, vpu, env and nef. (B) Predicted sites of the TRBP interaction with Dicer and the domain structure of TRBP and amino acid sequence of dsRBD3. TRBP is a dsRNA-binding protein consisting of 366 amino acid residues with three dsRBDs (dsRBD1, dsRBD2 and dsRBD3). dsRBD1 and dsRBD2 interact with dsRNA, while dsRBD3 is necessary for the interaction with Dicer. The complex structure of TRBP dsRBD3 and the ATPase/helicase domain of Dicer have been clarified by Wilson et al. (2015). From this structural analysis, the amino acid residues L326, V336, H338 and Y358 (pink circles in sequence) in TRBP were expected to be important for the interaction with Dicer. The interaction of these four amino acids (pink) in TRBP protein (green) with Dicer (blue) are in the crystal structure showing Dicer-TRBP interface (PDB, 4WYQ). The boxes i–iii indicate the region containing these amino acids (pink) and the boxes on the right are enlarged structures. (C) Analysis of the interaction between wild-type or mutant TRBPs with Dicer via immunoprecipitation (IP). TRBP^−/−^ HeLa cells were transfected with each expression plasmid (FLAG-tagged TRBP-WT, TRBP-dsRBDΔ3, TRBP-L326A, TRBP-V336A/H338A, TRBP-Y358A, TRBP-L326A/V336A/H338A/Y358A, TRBP-L326A/V336A/H338A, TRBP-V336A/V336A/H338A/Y358A, TRBPV336A/H338A/Y358A and TRBP-L326A/Y358A) and control Luc, and immunoprecipitated with anti-FLAG antibody. Finally, the TRBP–Dicer interaction was detected using anti-Dicer antibody. Asterisks indicate non-specific band.

Human immunodeficiency virus-1 (HIV-1) is the causative virus of acquired immunodeficiency syndrome (AIDS) (Barre-Sinoussi et al., 1983; Gallo et al., 1984). The virus expresses nine proteins. They are capable of evading the immune response (Pawlak and Dikeakos, 2015) and protecting infected cells from apoptosis (Selliah et al., 2003). Transactivating response (TAR) RNA-binding protein (TRBP) was first identified as a factor that binds to the stem-loop TAR RNA region of long terminal repeats (LTRs) on both the 5′- and 3′-ends of HIV-1 (Gatignol et al., 1991) (Fig. 1A). It has been demonstrated that translation of TAR RNA from the 5′-end is suppressed by its secondary structure (Dorin et al., 2003). However, when TRBP binds to TAR RNA, this inhibitory action on translation is relieved by an unknown mechanism (Dorin et al., 2003). An independent study revealed that Dicer, a double-stranded RNA (dsRNA)-cleaving enzyme, produces a ∼22 nucleotide (nt) microRNA (miRNA) called TAR miRNA via excision of the stem-loop-structured TAR RNA (Klase et al., 2007, 2009; Bennasser et al., 2004; Ouellet et al., 2008; Wagschal et al., 2012). The full-length viral RNA (Berkhout et al., 1989) and the short ∼60 nt non-processive TAR RNA with stem-loop structure, a substrate for the production of TAR miRNA (Harwig et al., 2016), are transcribed from the 5′ LTR. TAR miRNA can inhibit viral replication (Li et al., 2016) and protect infected cells from apoptosis by downregulating cellular genes, such as G-protein-coupled kinase-interacting protein 2 (GIT2) and immediate early response 3 (IER3) (Klase et al., 2009). However, how Dicer recognizes TAR RNA for processing remains unknown.

TRBP is a protein with three dsRNA binding domains (dsRBD1–3) (Fig. 1B). dsRBD1 and dsRBD2 are canonical dsRBDs essentially necessary for interaction with dsRNA, while dsRBD3 is a non-canonical dsRBD lacking RNA-binding residues and instead interacts with other proteins, including Dicer (Laraki et al., 2008; Daniels et al., 2009; Takahashi et al., 2013). In the RNA interference (RNAi) pathway, long dsRNA is first processed by Dicer into small interfering RNA (siRNA) (Hutvágner et al., 2001; Ketting et al., 2001; Kim et al., 2005). siRNA is a part of the RNA-induced silencing complex (RISC)-loading complex (RLC) with Dicer, Argonaute (Ago) and TRBP or its homologous gene, a protein kinase R (PKR) activator (PACT), as the main components (Tomari et al., 2004; Maniataki and Mourelatos, 2005; Liu et al., 2012). TRBP interacts with Dicer via dsRBD3 and is considered to enhance the cleavage activity of long dsRNA by Dicer and loading onto the RLC (Chendrimada et al., 2005; Haase et al., 2005). siRNA unwinds into single-stranded RNA, and the RNA strand that functions as a guide remains in the Ago protein to form RISC. The target mRNA base-paired with the guide RNA in the RISC is cleaved to suppress gene expression. In a similar pathway, RNA silencing is induced by small non-coding RNAs called miRNAs, which are encoded by genomic DNA and first transcribed as primary miRNAs (pri-miRNAs). Subsequently, pri-miRNAs are processed by the Drosha and DiGeorge Syndrome Critical Region 8 (DGCR8) proteins into ∼65 nt precursor miRNAs (pre-miRNAs) with stem-loop structures (Lee et al., 2003; Han et al., 2004; Denli et al., 2004; Gregory et al., 2004; Landthaier et al., 2004), which are transported to the cytoplasm by Exportin-5 (Yi et al., 2003; Lund et al., 2003; Kim, 2004). In the cytoplasm, pre-miRNAs undergo processing by Dicer into mature miRNAs (Hutvágner et al., 2001; Ketting et al., 2001; Winter et al., 2009; Grishok et al., 2001; Ha and Kim, 2014; Jinek and Doudna, 2009). Although Dicer alone can process pre-miRNA into mature miRNA (Chendrimada et al., 2005; Zhang et al., 2004; Provost et al., 2002), it can form a heterodimer with the Dicer-interacting dsRNA binding protein, TRBP or PACT, in human cells (Chendrimada et al., 2005; Kok et al., 2007; Taylor et al., 2013). The processing of pre-miRNA into mature miRNA is significantly enhanced by TRBP (Chendrimada et al., 2005; Haase et al., 2005; Chakravarthy et al., 2010). Single-molecule fluorescence techniques revealed that TRBP scans on dsRNA by an independent movement of two dsRBDs, dsRBD1 and dsRBD2, in which the diffusion distance is determined by the length of a linker domain that connects these two dsRBDs (Wang et al., 2015; Koh et al., 2016). Such a diffusion process functions as the dsRNA scanning mode of TRBP to stall off a physical barrier or bulky secondary structures (Koh et al., 2016), and TRBP recognizes dsRNA substrate with high affinity (Takahashi et al., 2013) discriminating specific structural characteristics (Takahashi et al., 2018) regardless of the end structure of the RNA. Then, TRBP positions the 3′-end of dsRNA onto the PAZ domain of Dicer to verify the authenticity of the substrate (Fareh et al., 2016). The substrate dsRNA lacking the 2-nt 3′-overhang is quickly released by TRBP, whereas the pre-miRNA with the 2-nt 3′-overhang is transferred to Dicer for cleavage. The PAZ domain of Dicer recognizes the 2-nt 3′-overhang of the pre-miRNA, and the region from the PAZ to RNase III domain acts as a molecular ruler to define the miRNA size (Yan et al., 2003; Macrae et al., 2006; MacRae et al., 2008; Tian et al., 2014). Thus, the Dicer-TRBP interaction is critical for efficient RNA processing among a large amount of cellular RNAs. In the downstream phase of RNA silencing, it was shown that the recruitment of miRNA to Ago protein also requires these Dicer-interacting proteins (MacRae et al., 2008). However, Dicer is not always necessary for processing of miRNA, and Dicer-independent processing of the pre-miRNAs has also been reported (Cifuentes et al., 2010; Yang et al., 2010; Cheloufi et al., 2010).

During RNAi/RNA silencing, TRBP functions in association with Dicer. In this study, we investigated whether Dicer collaboratively functions with TRBP in the inhibition of translational suppression of HIV-1 TAR RNA and whether Dicer is associated with TRBP in the TAR miRNA cleavage process.

## RESULTS

### Identification of TRBP amino acid residues essential for interaction with Dicer

TRBP was initially isolated for its ability to bind with TAR RNA, which is a stem-loop RNA in the R region of the HIV-1 LTR (Gatignol et al., 1991) (Fig. 1A). It has been shown that TRBP promotes translation of TAR-containing RNAs (Dorin et al., 2003). An independent study reported that TAR RNA is cleaved by Dicer in the stem region to produce a ∼22 nt TAR miRNA (Klase et al., 2007). However, during RNA silencing, TRBP functions to promote RNAi activity by interacting with Dicer (Chendrimada et al., 2005; Haase et al., 2005). TRBP has three dsRBDs, and interacts with dsRNA via dsRBD1 and dsRBD2 (Daniels et al., 2009; Takahashi et al., 2013) and with Dicer via dsRBD3 (Gatignol et al., 1991) (Fig. 1B). Since TRBP can bind to both Dicer and TAR RNA, TRBP-mediated translational enhancement of TAR-containing RNA and Dicer-mediated TAR miRNA production may occur through the TRBP–Dicer interaction. To verify this hypothesis, we constructed expression constructs of TRBP mutants that were unable to interact with Dicer.

According to structural analysis of the interaction between the dsRBD3 of TRBP and the ATPase/helicase domain of Dicer (Wilson et al., 2015), leucine at position 326 (L326), valine at position 336 (V336), histidine at position 338 (H338) and tyrosine at position 358 (Y358) in TRBP were predicted to be important residues in the interaction with Dicer (Fig. 1B). Therefore, we constructed the following FLAG-tagged TRBP expression plasmids: pFLAG-TRBP-L326A (L326 was replaced with alanine); pFLAG-TRBP-Y358A (Y358 was replaced with alanine); pFLAG-TRBP-V336A/H338A (V336 and H338 were each replaced with alanine); pFLAG-TRBP-L326A/Y358A (L326 and Y358 were each replaced with alanine); pFLAG-TRBP-L326A/V336A/H338A (L326, V336 and H338 were each replaced with alanine); pFLAG-TRBP-V336A/H338A/Y358A (V336, H338 and Y358 were each replaced with alanine); and pFLAG-TRBP-L326A/V336A/H338A/Y358A (L326, V336, H338 and Y358 were each replaced with alanine).

Using the plasmid-expressing FLAG-tagged wild-type TRBP (TRBP-WT) and TRBP mutants, we identified the amino acids essential for the interaction with Dicer using immunoprecipitation. Each plasmid was transfected into human HeLa cells in which TRBP was knocked out via the CRISPR/Cas9 system (Takahashi et al., 2018). Immunoprecipitation of the TRBP protein was performed using anti-FLAG antibody, and western blotting with anti-Dicer antibody was performed to detect the interaction with Dicer (Fig. 1C). As a negative control, pFLAG-TRBP-dsRBDΔ3 plasmid with deletion of dsRBD3, which is necessary for the interaction with Dicer, and a plasmid expressing the firefly *luciferase* gene (Luc) were used. All TRBP mutant-expressing constructs were successfully expressed, and Dicer was immunoprecipitated with TRBP-WT but not with TRBP-dsRBDΔ3. The interaction between TRBP and Dicer was not changed or weakly attenuated by expression of TRBP-L326A, TRBP-V336A/H338A and TRBP-Y358A, whereas TRBP-L326A/V336A/H338A/Y358A, TRBP-L326A/V336A/H338A, TRBP-V336A/H338A/Y358A and TRBP-L326A/Y358A markedly reduced the interaction with Dicer. These results suggest that all four tested TRBP residues (i.e. L326, V336, H338 and Y358) may partially contribute to its interaction with Dicer, but mutations of L326 and Y358 alone are sufficient to prevent this interaction.

### Establishment of Flp-In 293 cells expressing TRBP mutants lacking the interaction with Dicer

**Fig. 2.**
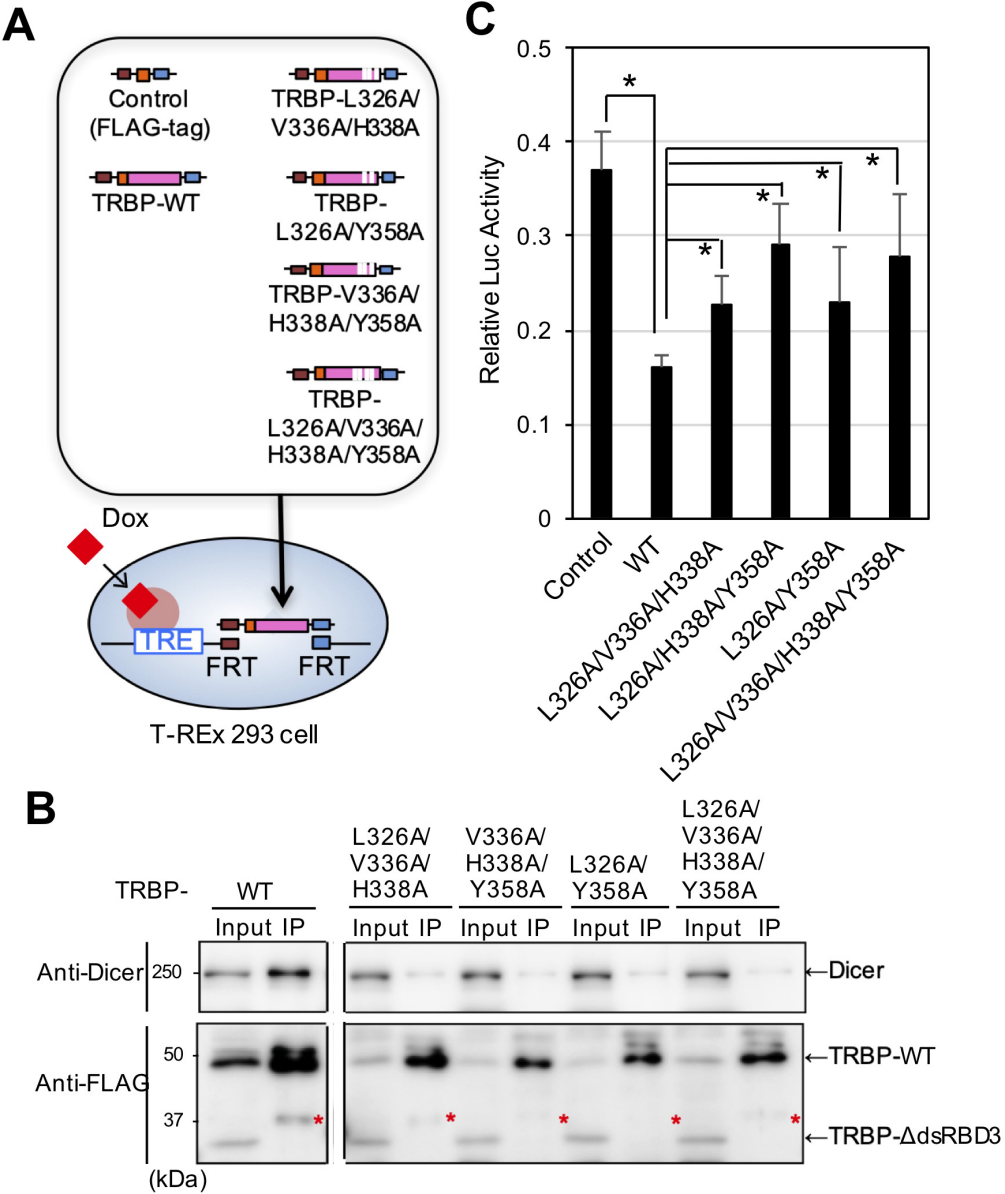
**Establishment of the Flp-In 293 cell line expressing wild-type and mutant TRBP.** (A) T-REx 293 cells contain a TRE promoter capable of inducing downstream gene expression with tetracycline or Dox addition. Using this system, cell lines introduced with FLAG-tag alone (Control), FLAG-tagged TRBP-WT, TRBP-L326A/V336A/H338A, TRBPL326A/Y358A, TRBP-V336A/H338A/Y358A or TRBP-L326A/V336A/H338A/Y358A were established. (B) Confirmation of TRBP expression in Flp-In 293 cells. The established Flp-In 293 cells introducing each plasmid were cultured in medium containing Dox, and western blotting and IP using anti-FLAG antibody were performed. (Left) All incorporated FLAG-tagged TRBP genes were successfully expressed. The interaction with Dicer was attenuated in all cell lines except for cells with TRBP-WT. Asterisk indicates non-specific band. (Right) The intensities in the western blot bands in the left panels were measured using ImageJ, and showed the relative levels of immunoprecipitated Dicer compared to the immunoprecipitated TRBP and its mutants. These results were obtained under nearly identical conditions to Fig. 1C. Asterisks indicate non-specific band. (C) Effect on RNAi activity of TRBP-WT and its mutant TRBPs. Flp-In 293 cells expressing each TRBP were transfected with firefly *luciferase* and *Renilla luciferase* expression plasmids with the shRNA expression plasmid against firefly luciferase (pSUPER-FL774). Cells were harvested after 24 h and luciferase luminescence intensity was measured. The luciferase activity of each cell type was normalized using the value obtained by transfection of control shRNA expression plasmid against green fluorescent protein (pSUPER-GY441) in control cells. The data represent the mean±s.d. of two independent experiments; each experiment was carried out using three wells. Student's *t*-test, *P<0.01.

In the Flp-In 293 cell line, the tetracycline response element (TRE) promoter is incorporated into the genome, and any gene can be introduced into the downstream flippase recognition target (FRT) site via homologous recombination with flippase (FLP) recombinase (Fig. 2A). We established Flp-In 293 cells expressing FLAG-tag alone as control cells, and those expressing FLAG-tagged TRBP-WT (Takahashi et al., 2013) and TRBP mutants, including FLAG-tagged TRBP-L326A/V336A/H338A, TRBP-V336A/H338A/Y358A, TRBP-L326A/Y358A and TRBP-L326A/V336A/H338A/Y358A, were also established. Two days after doxycycline (Dox) addition, western blotting was performed using anti-FLAG antibody. The expression of all introduced genes was observed (Fig. 2B, left panel). Dicer was immunoprecipitated with TRBP-WT, and the interaction with Dicer by the TRBP mutants TRBP-L326A/V336A/H338A, TRBP-V336A/H338A/Y358A, TRBP-L326A/Y358A and TRBP-L326A/V336A/H338A/Y358A was markedly attenuated (Fig. 2B), consistent with the results of the overexpression experiments (Fig. 1C). Thus, we considered Flp-In 293 cell lines capable of inducing mutant TRBPs lacking the interaction with Dicer were established.

### Effect of the TRBP–Dicer interaction on RNAi activity

To investigate the effect of the TRBP–Dicer interaction on RNAi activity, a dual luciferase reporter assay was carried out using Flp-In cells containing control FLAG-tag alone, TRBP-WT, TRBP-L326A/V336A/H338A, TRBP-V336A/H338A/Y358A, TRBP-L326A/Y358A and TRBP-L326A/V336A/H338A/Y358A. The cells were cultured in medium containing Dox for 24 h to induce TRBP expression and transfected with the firefly *luciferase* (pGL3-control) and *Renilla luciferase* (pRL-SV40) expression plasmid along with the short hairpin RNA (shRNA) expression plasmid against the firefly *luciferase* gene. The cells were harvested 24 h after transfection, and the intensity of luciferase luminescence was measured and calculated relative luciferase activity (firely luciferase activity/Renilla luciferase activity) (Fig. 2C). In this experiment, we used the shRNA expression plasmid instead of siRNA. Because shRNA undergoes Dicer-mediated cleavage, the TRBP–Dicer interaction was considered to show marked effects with shRNA compared to siRNA, which does not undergo this process. The relative luciferase activity was decreased in TRBP-WT-expressing cells compared to control cells, since luciferase activity is suppressed by RNAi, consistent with previous reports (Takahashi et al., 2014). Furthermore, the repression activities in all the cells expressing TRBP mutants lacking the interaction with Dicer (i.e. TRBP-L326A/V336A/H338A, TRBP-V336A/H338A/Y358A, TRBP-L326A/Y358A and TRBP-L326A/V336A/H338A/Y358A) were significantly weaker compared to TRBP-WT-expressing cells.

### Translational regulation of TAR-containing RNA via the TRBP–Dicer interaction

**Fig. 3.**
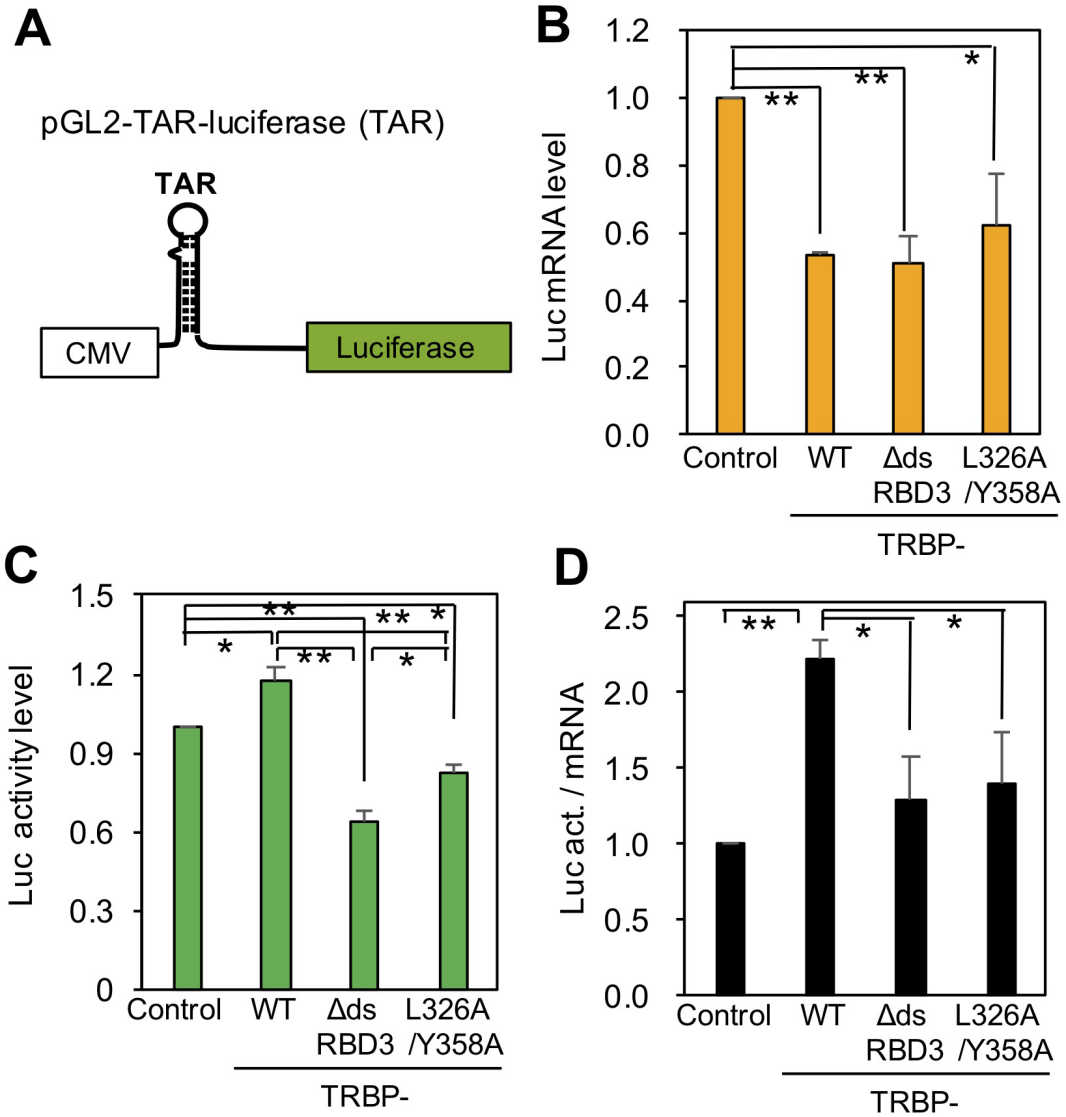
**Translational regulation of HIV-1 TAR RNA by TRBP.** (A) Schematic representation of pGL2-TAR-Luciferase expression plasmid. pGL2-TAR-Luciferase contained the 5′-LTR of the HIV-1 genome containing TAR downstream of the CMV promoter and fused with the firefly *luciferase* gene. (B) Luciferase mRNA levels in Flp-In 293 cells determined with real-time PCR. Flp-In 293 cells with control FLAG-tag alone, FLAG-tagged TRBP-WT, TRBP-dsRBDΔ3 or TRBP-L326A/Y358A were transfected with pGL2-TAR-Luciferase along with pRL-SV40. The cells were recovered after 24 h, and the amount of luciferase mRNA was measured. mRNA levels were normalized using the mRNA level of endogenous GAPDH. The amount of *luciferase* mRNA in the control cells transfected with pGL2-TAR-Luciferase was set as 1. (C) Luciferase activity in Flp-In 293 cells based on the luciferase reporter assay system. Using cells transfected as described in B, the intensity of luciferase luminescence was measured. The relative luciferase activity in the control cells transfected with pGL2-TAR-Luciferase was set as 1. (D) Luciferase activities shown in C were normalized by their mRNA amounts in B. The luciferase activity normalized by mRNA amount in the control cells transfected with pGL2-TAR-Luciferase was set as 1. The data represent the mean±s.d. of three independent experiments. Student's *t*-test, **P<0.01, *P<0.05.

To investigate the effects of the TRBP–Dicer interaction on the regulation of TAR RNA-mediated translational activity, the translational activity of TAR-containing RNA in Flp-In 293 cells expressing TRBP-WT, TRBP-dsRBDΔ3 or mutant TRBP lacking the interaction with Dicer (i.e. TRBP-L326A/Y358A) was evaluated using the luciferase reporter with TAR upstream of the *luciferase* gene (pGL2-TAR-luciferase) (Dorin et al., 2003) (Fig. 3A). TRBP-L326A/Y358A plasmid was selected as a typical Dicer-interaction deficient mutant, since only two amino acids were necessary for lacking Dicer interaction. The plasmid was transfected into Flp-In 293 cells with the control expression construct of *Renilla luciferase* and cultured in medium containing Dox for 24 h. Total RNA was purified from cells and real-time PCR was performed to measure *luciferase* gene mRNA levels (Fig. 3B) and protein levels based on luciferase activity (Fig. 3C).

*Luciferase* gene mRNA levels were significantly higher in control cells, but were 0.4–0.6-fold lower in cells expressing TRBP-WT, TRBP-dsRBDΔ3 and TRBP-L326A/Y358A (Fig. 3B). This means that transfection of TRBP-expression plasmids repressed luciferase transcription regardless of Dicer binding. Next, the intensity of luciferase luminescence was measured (Fig. 3C). It was clearly observed that the relative luciferase activity was significantly increased in the cells expressing TRBP-WT compared to the cells expressing TRBP-dsRBDΔ3 or TRBP-L326A/Y358A. In order to examine the translational activity of TRBP protein, relative luciferase activity was normalized by mRNA amount (Fig. 3D). The translational activity was significantly increased by TRBP-WT but not by TRBP-dsRBDΔ3 or TRBP-L326A/Y358A. These results suggest that the translational activity of TAR-containing RNA is strongly enhanced by TRBP–Dicer interactions. Thus, it was inferred that TRBP binds to the TAR RNA and recruits Dicer by direct TRBP-Dicer interaction, and Dicer is expected to cleave out the stem-loop-structured TAR RNA, which is the translational suppressor, to facilitate translation of TAR-containing RNA.

### TAR miRNA processing via the TRBP–Dicer interaction

**Fig. 4.**
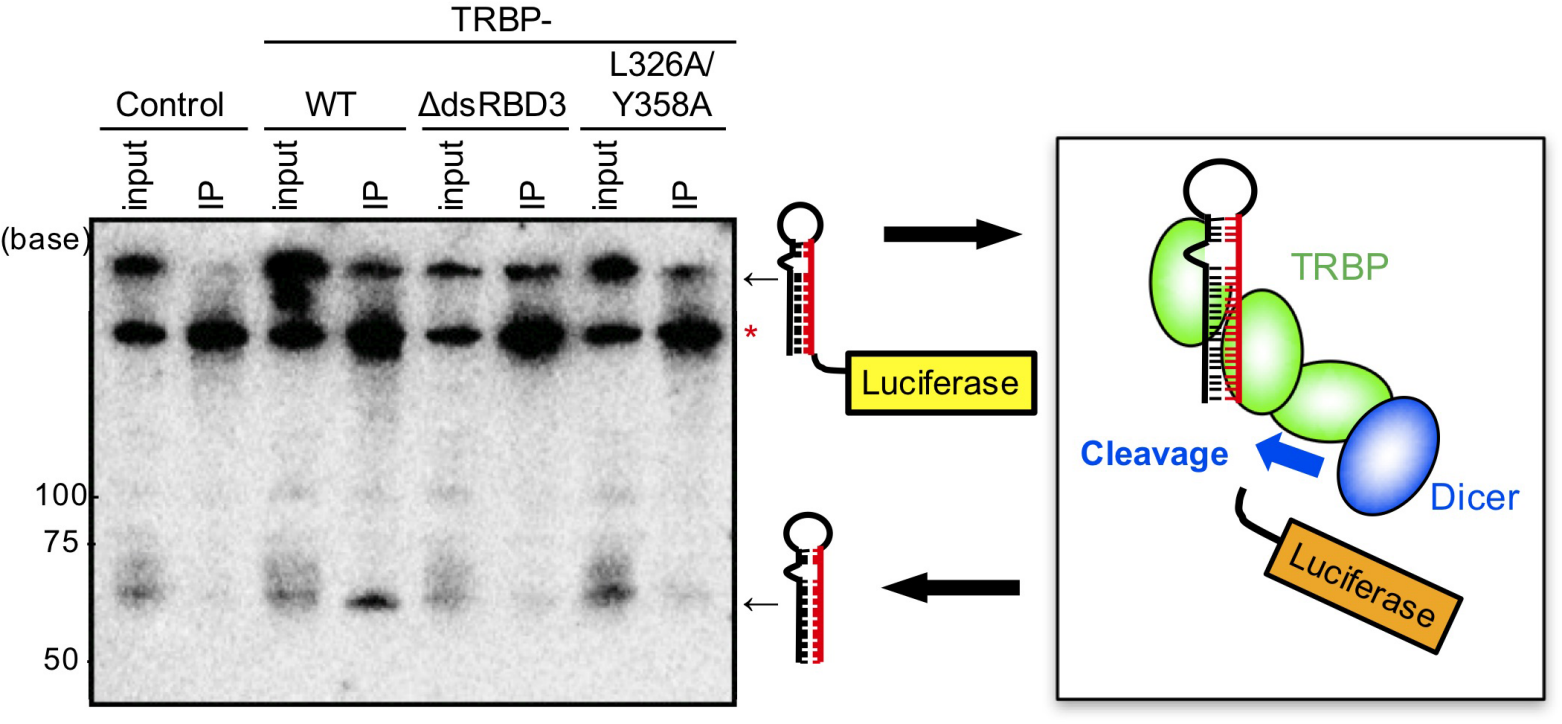
**Northern blot analysis of the excision of TAR miRNA.** Flp-In 293 cells with control FLAG-tag alone, FLAG-tagged TRBP-WT, TRBP-dsRBDΔ3 or TRBP-L326A/Y358A were transfected with pGL2-TAR-Luciferase, and IP was performed with anti-FLAG antibody. Northern blotting was performed using purified total RNA from input samples and IP samples with a probe for detecting the 3′ stem region of TAR RNA (red). A band of approximately 60 bases corresponding to the cleaved stem-loop-structured TAR RNA was observed in the IP sample of TRBP-WT, but not in those of TRBP-dsRBDΔ3 or TRBPL326A/Y358A. The right figure presents a model of TAR RNA cleavage via the TRBP–Dicer interaction. Asterisk indicates non-specific band.

The non-processive transcript from the HIV-1 LTR promoter produces short ∼60 nt TAR miRNA precursor with a stable stem-loop structure is cleaved by Dicer into TAR miRNA (Harwig et al., 2016). However, although translational suppression by TAR RNA is observed in the processive transcript, whether the processive transcript is also cleaved by Dicer or not is unclear. To examine the contribution of TRBP to the cleavage from the processive transcript into stem-loop-structured TAR RNA, which may function as a TAR miRNA precursor, in collaboration with Dicer Flp-In 293 cells expressing FLAG-tagged TRBP-WT and its mutants were cultured in medium containing Dox to induce TRBP expression. Then, cells were transfected with pGL2-TAR-luciferase, harvested after 24 h, and immunoprecipitated with anti-FLAG antibody. RNA interacting with TRBP was purified and northern blotting was performed to detect the cleavage of stem-loop-structured TAR RNA. A DNA probe with a complementary sequence to the 3′ stem region of TAR miRNA precursor was labelled with γ-^32^P-ATP at the 5′-end, and the TAR miRNA precursor was detected by northern blotting. In the immunoprecipitated sample of TRBP-WT, a signal approximately 60-nt long band corresponding to a TAR miRNA precursor, estimated from the length, was detected (Fig. 4). However, in the immunoprecipitated samples of TRBP-dsRBDΔ3 or TRBP-L326A/Y358A, no signals considered to be TAR miRNA precursors were detected. A somewhat similar result was obtained when the DNA probe with a complementary sequence to the 5′ stem region of TAR miRNA precursor was used (Fig. S1). The small amounts of possible TAR miRNA precursors detected in input lanes but not in immunoprecipitated (IP) lanes in all samples may be cleaved products of endogenous TRBP and Dicer. These results suggest that the TAR miRNA precursor from the processive transcript is excised by Dicer dependent on TRBP, which binds to the TAR RNA region and may recruit Dicer. The mature TAR miRNA band, about 20-nt long, was not detected in our northern blot, even in the input lanes (Fig. 4; Fig. S1). It was speculated that the endogenous TRBP-Dicer could not cleave the exogenous TAR-luciferase sufficiently. Furthermore, mature TAR miRNA could not be detected in the IP lane of TRBP-WT by northern blot analysis, likely because mature single-stranded miRNAs were released from TRBP and were not immunoprecipitated.

### TNFα- and cycloheximide-induced apoptosis is regulated by the TRBP–Dicer interaction

TAR miRNA protects virus-infected cells from apoptosis by downregulating apoptosis-related genes (Klase et al., 2009). Based on northern blotting (Fig. 4), the TAR miRNA precursor was excised from the TAR RNA region in the presence of TRBP-WT, but not the TRBP mutant lacking the Dicer interaction. Thus, the TRBP–Dicer interaction was considered to be necessary for excision of TAR miRNA precursor, and probably also TAR miRNA.

**Fig. 5.**
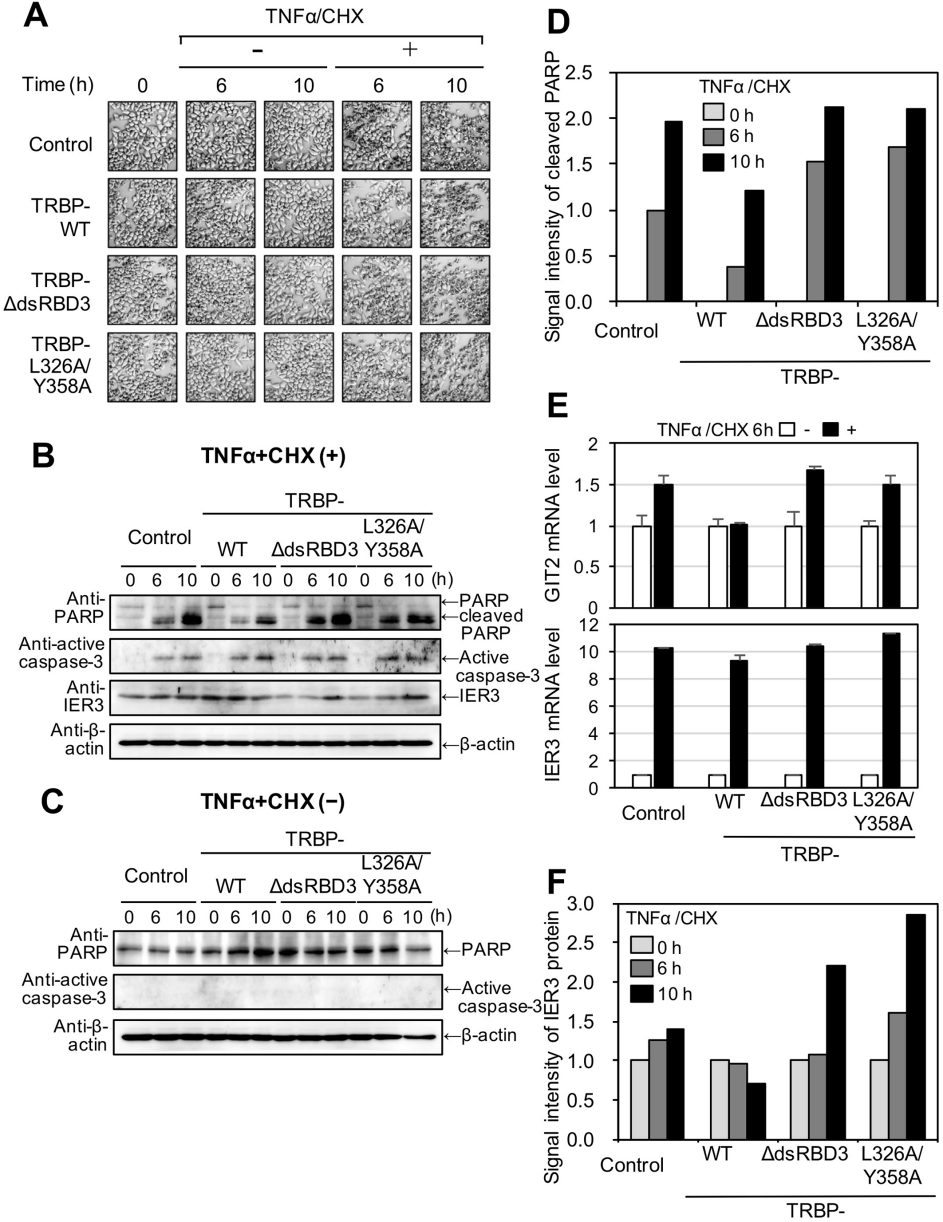
**Regulation of apoptosis by the TRBP–Dicer interaction.** (A) Morphological changes in TRBP^−/−^ HeLa cells transfected with pGL2-TAR-Luciferase along with each expression plasmid of control FLAG-tag alone, FLAG-tagged TRBP-WT, TRBPdsRBDΔ3 or TRBP-L326A/Y358A at 0, 6 and 10 h after treatment with or without TNFα/CHX. (B,C) Western blots of the apoptosis marker proteins PARP, caspase-3 and IER3 at 0, 6 and 10 h after treatment with TNFα/CHX (B) or without TNFα/CHX (C). β-actin was used as control. Processing of PARP or caspase-3 was confirmed in cells with induced apoptosis following TNFα/CHX treatment (B), but processing was not observed in cells without TNFα/CHX (C). (D) Quantification of cleaved PARP protein at 6 and 10 h after addition of TNFα/CHX. The western blot result shown in B was quantified using ImageJ. The proportions of processed PARP relative to the total amount of PARP protein including full-length and processed PARP were calculated as a percentage. The amount of cleaved PARP in control cells at 6 h after TNFα/CHX treatment was set as 1. (E) GIT2 and IER3 mRNA levels measured by real-time PCR. mRNA levels were normalized to the amount of tublinβ mRNA. The amount of GIT2 or IER3 mRNA at 6 h without TNFα/CHX treatment was set as 1. (F) Quantification of IER3 protein at 0, 6 and 10 h after the addition of TNFα/CHX. The western blot results shown in C were quantified using ImageJ. The amount of IER3 protein at 0 h was set as 1. Two independent experiments were performed, and their results were almost similar. The results shown in B and D were used for quantification in D, E and F.

Next, TRBP^−/−^ HeLa cells were transfected with pGL2-TAR-luciferase with each TRBP expression plasmid (FLAG-tagged TRBP-WT, TRBP-dsRBDΔ3 or TRBP-L326A/Y358A). After 24 h, cells were plated in 12-well culturing plates. After an additional 24 h, the medium was replaced with medium containing tumour necrosis factor (TNF)-α and cycloheximide (CHX), which induce apoptosis (Miura et al., 1995). The cells were photographed under a microscope at 0, 6 and 10 h after the addition of TNFα and CHX (Fig. 5A). In cells transfected with any of the plasmids, typical apoptotic morphological changes were observed: cells appeared shrunken at 6 h, then they detached from the dishes, and floating fragmented cells were observed at 10 h. However, no clear differences in morphological changes were observed among the transfected plasmids.

Cells were collected at 6 and 10 h following TNFα/CHX treatment, and western blotting with anti-FLAG, anti-poly[ADP-ribose] polymerase (PARP), or anti-caspase-3 antibodies was performed (Fig. 5B,C). Apoptotic markers of PARP and caspase-3 (Germain et al., 1999; Boulares et al., 1999) are processed during apoptosis. The results showed that TNFα/CHX treatment induced cleavage of PARP and caspase-3 (Fig. 5B) regardless of which plasmid was transfected. However, PARP and caspase-3 were not cleaved in cells not treated with TNFα/CHX (Fig. 5C), indicating that apoptosis was induced by TNFα/CHX treatment in TRBP^−/−^ HeLa cells. Next, the ratio of cleaved PARP to the total amount of cleaved and uncleaved PARP protein was determined at 6 and 10 h after TNFα/CHX treatment. The amount of cleaved PARP in cells expressing TRBP-WT was low compared to that in control cells at 6 and 10 h after TNFα/CHX treatment, consistent with a previous report (Chukwurah and Patel, 2018), while their amounts in cells expressing TRBP-dsRBDΔ3 and TRBP-L326A/Y358A were higher than or comparable to the control (Fig. 5D). The results suggest that TRBP-WT interacts with endogenous Dicer to cleave TAR miRNA precursor, and TAR miRNA precursor or its cleaved product, TAR miRNA, functions to repress apoptosis. However, apoptosis was not repressed by TRBP-dsRBDΔ3 and TRBP-L326A/Y358A, since TAR miRNA precursor was not processed in these mutant cells, which could not recruit Dicer to the TAR region.

**Fig. 6.**
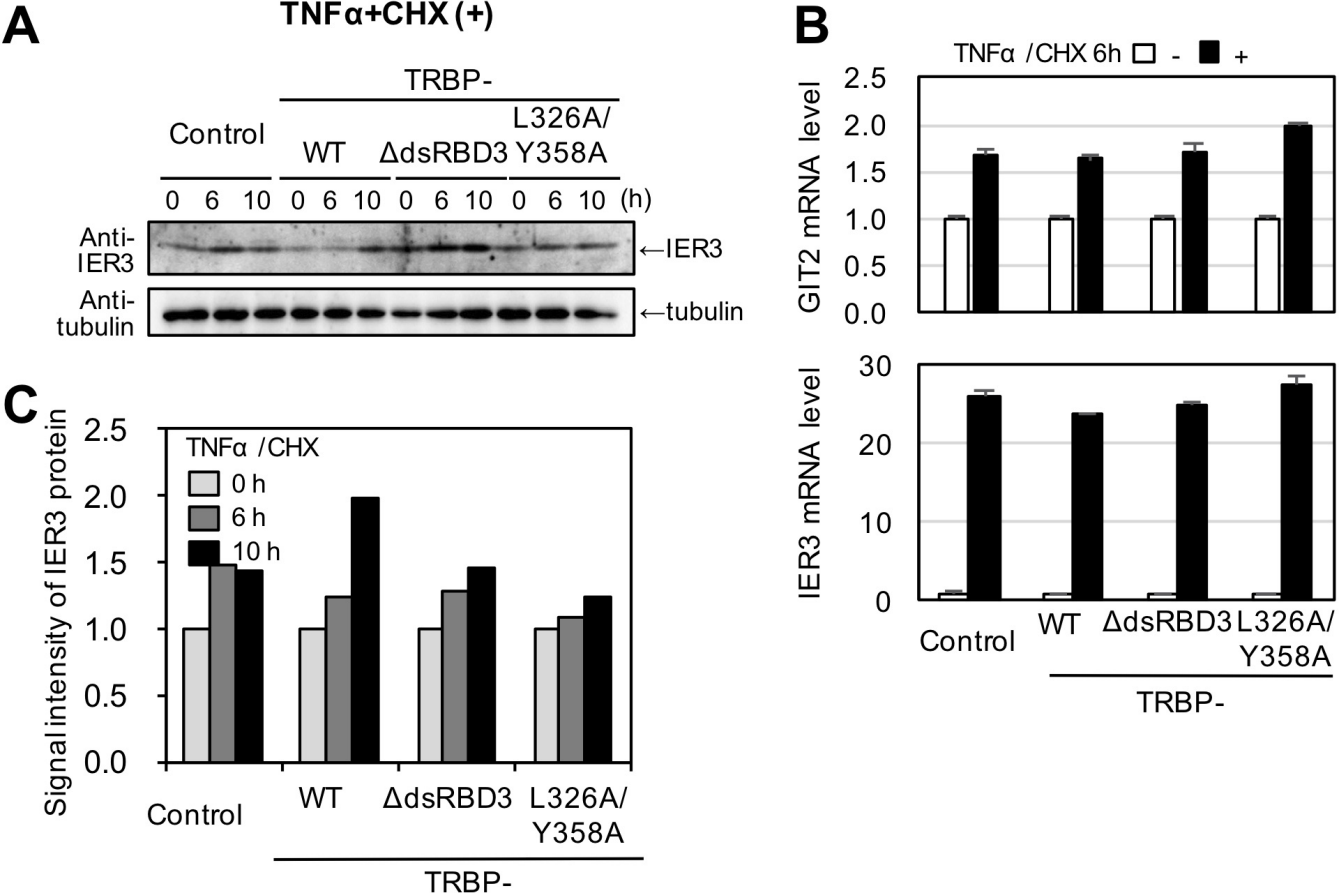
**Regulation of GIT2 or IER3 expression without TAR miRNA expression.** (A) Western blots of IER3 at 0, 6 and 10 h after treatment with TNFα/CHX. α-tubulin was used as control. (B) GIT2 and IER3 mRNA levels measured by real-time PCR. mRNA levels were normalized to the amount of tublinβ mRNA. The amount of GIT2 or IER3 mRNA at 6 h without TNFα/CHX treatment was set as 1. (C) Quantification of IER3 protein at 0, 6 and 10 h after the addition of TNFα/CHX. The western blot results shown in A were quantified using ImageJ. The amount of IER3 protein at 0 h was set as 1. Two independent experiments were performed, and their results were similar. The results shown in A were used for quantification in B and C.

Furthermore, mRNA levels of GIT2 and IER3 genes induced by apoptosis and inhibited by TAR miRNA (Klase et al., 2009) were measured with real-time PCR at 6 h after TNFα/CHX treatment (Fig. 5E). GIT2 mRNA levels were unchanged in cells expressing TRBP-WT, while their expression levels were increased in control TRBP^−/−^ HeLa cells and the cells expressing TRBP-dsRBDΔ3 and TRBP-L326A/Y358A (Fig. 5E). The result suggests that GIT2 mRNA expression is repressed by TAR miRNA precursor or TAR miRNA, which might be produced by TRBP-Dicer interaction. Meanwhile, IER3 mRNA levels were increased in all cells by the TNFα/CHX treatment. Then, the amount of IER3 protein was further measured. The results show that IER3 protein levels were slightly repressed by TRBP-WT expression, but increased by TRBP-dsRBDΔ3 or TRBP-L326A/Y358A expression (Fig. 5F). These results suggest that TAR miRNA precursor or TAR miRNA does not evidently repress mRNA expression in the case of IER3, but represses IER3 translation in a post-transcriptional manner when apoptosis is induced. Furthermore, we performed the experiments to confirm the contribution of TAR RNA using cells transfected with pGL2-ΔTAR-luciferase lacking TAR region with each TRBP expression plasmid (FLAG-tagged TRBP-WT, TRBP-dsRBDΔ3 or TRBP-L326A/Y358A). By treatment with TNFα/CHX, GIT2 mRNA levels were increased in all the cells transfected with pGL2-ΔTAR-luciferase regardless of co-transfected TRBP expression constructs (Fig. 6). Furthermore, the protein levels of IER3 in addition to its mRNA levels were also increased in all cells (Fig. 6). Overall, these results indicate that the TAR region is essential for the repression of GIT2 and IER3, suggesting that Dicer processes TAR miRNA precursor or TAR miRNA in the presence of TRBP and they may suppress the GIT2 mRNA level and IER2 protein level by post-transcriptional regulation.

## DISCUSSION

**Fig. 7.**
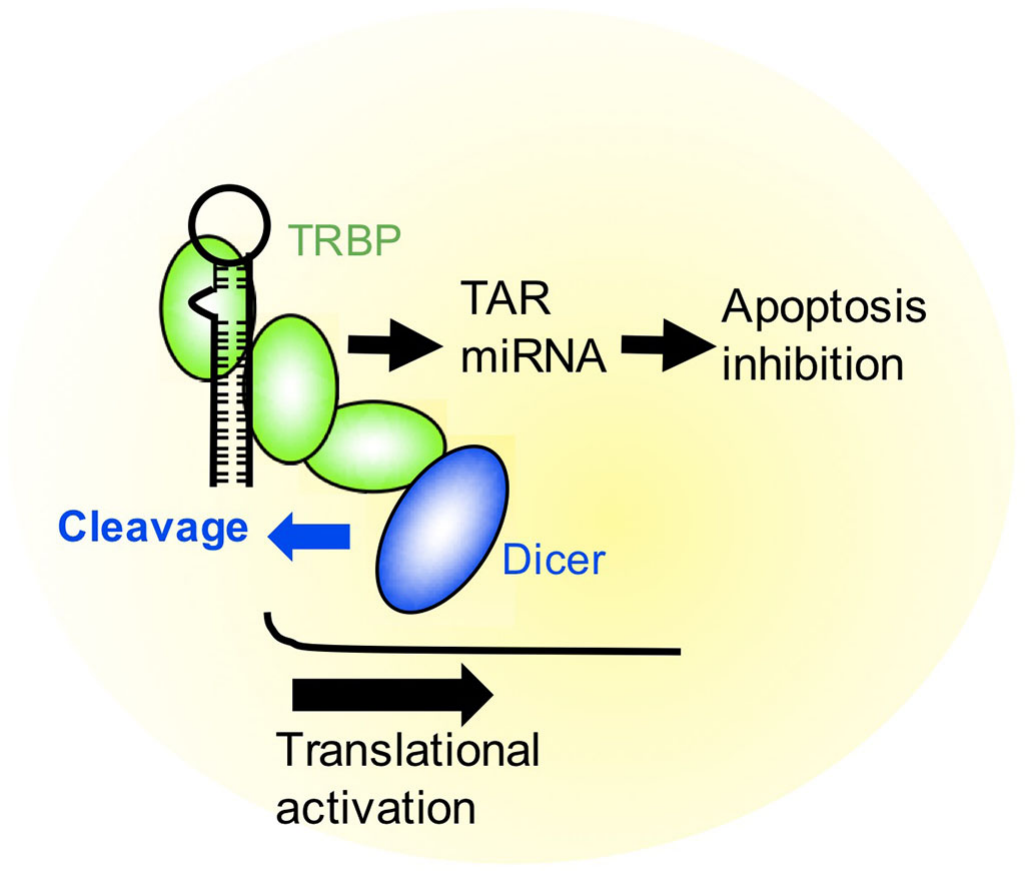
**Model of TRBP function in HIV-1 infection.** TRBP is essential for excision of TAR miRNA in addition to Dicer. Furthermore, Dicer is essential for translational activation of TAR RNA in addition to TRBP. Thus, the TRBP–Dicer interaction is necessary for both processing of TAR miRNA and translational activation of TAR-containing RNA.

TRBP functions to enhance RNAi activity by interacting with Dicer (Chendrimada et al., 2005; Haase et al., 2005). Upon HIV-1 infection, TRBP binds to the TAR RNA region of HIV-1 to repress translational inhibition and enhance translation (Gatignol et al., 1991). In this study, we successfully constructed TRBP mutants that could not interact with Dicer based on a structural study of the TRBP–Dicer interaction (Fig. 1). Using mutant TRBPs and cells expressing the constructs, we revealed that, in addition to TRBP, Dicer may be necessary for the repression of translational inhibition (Fig. 3). In this process, it is speculated that TRBP binds to the stem-loop-structured TAR RNA region and recruits the Dicer protein by association with TRBP dsRBD3 to excise the TAR miRNA precursor (Figs 4 and 7). TAR RNA-mediated translation is increased when the TAR structure is mutated or deleted (Dorin et al., 2003). Thus, the secondary structure of TAR may function to disturb the translation, then the cleaved RNA-excised TAR RNA might be able to initiate translation. However, how a cleaved HIV RNA recruits a ribosome is unknown, since methylated 5′ cap is necessary for ribosome loading.

In an independent study, Dicer has been reported to promote TAR miRNA processing from non-processive ∼60 nt TAR RNA (Klase et al., 2009). Our results suggested that Dicer is also associated for production of TAR miRNA precursor from the processive TAR RNA by interaction with TRBP (Fig. 4). Since it is generally accepted that Dicer cleaves pre-miRNA into mature miRNA in the cytoplasm (Hutvágner et al., 2001; Ketting et al., 2001; Winter et al., 2009; Grishok et al., 2001; Ha and Kim, 2014; Jinek and Doudna, 2009), the cleavage of the processive TAR RNA resulting in TAR miRNA precursor is not the canonical function of Dicer. Usually, pri-miRNA is cleaved into pre-miRNA by Drosha and DGCR8 in the nucleus (Lee et al., 2003; Han et al., 2004; Denli et al., 2004; Gregory et al., 2004; Landthaier et al., 2004), and Dicer cleaved pre-miRNA into mature miRNA in the cytoplasm (Klase et al., 2007, 2009; Bennasser et al., 2004; Ouellet et al., 2008; Wagschal et al., 2012).

PACT is a homologous gene of TRBP and also interacts with Dicer. Each of them can homodimerize, respectively, and TRBP can heterodimerize with PACT. PACT homodimerizes through two domains (dsRBD1 and dsRBD2) in Kok (2007) but through three domains in Laraki (2008). TRBP homodimerizes through two domains (dsRBD1 and dsRBD2), and *in vitro* siRNA binding experiments of TRBP showed that the *K_d_* values of homodimerization of siRNA-bound TRBP lacking dsRBD3 are higher compared to those of full-length TRBP (Takahashi et al., 2013). Thus, dsRBD3 may be important for homodimerization of these proteins. Furthermore, the mutagenesis of amino acid in PACT corresponding to L326 in TRBP disrupted their homodimerization (Heyam et al., 2017). Our result indicates that the mutated TRBP at L326 and Y358 cannot interact with Dicer (Figs 1 and 2), suggesting that Dicer-binding and homodimerize interfaces overlap in these proteins. Thus, TRBP protein binding to stem-loop-structured TAR RNA may be considered to be monomer as shown in Fig. 7.

This is the first report to suggest that the TRBP–Dicer interaction is important for TAR RNA translation, TAR miRNA precursor excision and the regulation of HIV-1 genome expression. However, it is impossible to exclude the possibility that the factor(s) other than Dicer interacting with TRBP may be involved in the regulation of HIV-1 expression. In Fig. 3B, *luciferase* mRNA levels were significantly higher in control cells compared to those in the cells expressing TRBP-WT, TRBP-dsRBDΔ3 and TRBP-L326A/Y358A, suggesting that TRBP repressed luciferase transcription via other interacting proteins regardless of Dicer binding. Furthermore, TRBP-WT increased luciferase activity to 1.2-fold compared to the control (Fig. 3C). However, Dorin et al. (2003) showed that the same plasmid increased luciferase translation to 2∼4-fold in PKR ^−/−^ cells. PKR is a serine/threonine protein kinase that is activated by interferons, dsRNAs, cytokine, growth factor and stress signals (Garcia-Ortega et al., 2017), and inhibits translation and promotes apoptosis through the substrates, including eukaryotic translation initiation factor 2 alpha (eIF2α), protein phosphatase 2A (PP2A), inhibitor of nuclear factor kappa-B(IκB) kinase (IKK) and downstream effectors (Garcia et al., 2006). Phosphorylated eIF2α inhibits translation initiation but activates some selected proteins critical for cell survival (Jiang, et al., 2004). Thus, if PKR can be associated with TRBP-WT and also TRBP mutants lacking Dicer interaction, the luciferase translation may be repressed by PKR interaction with TRBP.

TRBP may bind to the TAR region and induce the excision of TAR miRNA precursor and probably also TAR miRNA by interacting with Dicer when the HIV-1 virus infects host cells. Then, the TRBP–Dicer interaction may prevent apoptosis of infected cells by RNA silencing and activates HIV-1 replication, likely by removing the steric hindrance against translation of the TAR region. Therefore, TRBP and Dicer may function to be endogenous regulators of HIV-1 replication in host cells when these factors are abundant in the cells.

## MATERIALS AND METHODS

### Cell culture

Human cervical cancer cell-derived HeLa cells, kindly supplied from Hiroyuki Sasaki at Kyushu University, Japan, were cultured in Dulbecco's Modified Eagle Medium (DMEM) supplemented with 10% inactivated fetal bovine serum (FBS) at 37°C with 5% CO_2_. Human embryonic kidney cell-derived Flp-In T-REX 293 cells (Life Technologies) were cultured in DMEM containing 10% heat-inactivated Tet-system approved FBS (Clontech) without tetracycline and 10 ng/ml of Hygromycin B at 37°C with 5% CO_2_.

### Preparation of mutant TRBP expression constructs

A wild-type TRBP expression construct (pcDNA5-TRBP-WT) containing a full length TRBP (Takahashi et al., 2013) at the *Nhe*I/*Hind*III sites of pcDNA5-FRT-TO-FLAG/HA-B (Okada-Katsuhata et al., 2012) was used as a template. A mutant TRBP-L326A expression construct, in which leucine at position 326 was replaced with alanine, was generated using primers (Table S1) and KOD-Plus-Mutagenesis Kit (TOYOBO) by site-directed mutagenesis method. In addition, a single amino acid mutant of TRBP-Y358A, double amino acids mutants of TRBP-L326A/V336A and TRBP-V336A/H338A, triple mutants of TRBP-V336A/H338A/Y358A and TRBP-L326A/V336A/H338A and a quadruple mutant of TRBP-L326A/V336A/H338A/Y358A were also constructed by the same procedures using primers (Table S1), and designated as pcDNA5-TRBP-L326A, pcDNA5-TRBP-Y358A pcDNA5-TRBP-L326A/V336A, pcDNA5-TRBP-V336A/H338A, pcDNA5-TRBP-V336A/H338A/Y358A, pcDNA5-TRBP-L326A/V336A/H338A and pcDNA5-TRBP-L326A/V336A/H338A/Y358A, respectively. The nucleotide sequences of generated expression constructs were confirmed by sequencing.

### Establishment of Flp-In cells expressing wild-type and mutant TRBPs

Four millilitres of cell suspension of Flp-In T-REX 293 cells at 1×10^5^ cells/ml were inoculated into each well of six-well cell culture plates (Sumitomo Bakelite). Two-hundred microliters of OPTI-MEM containing 1 μg wild-type TRBP (pcDNA5-TRBP-WT) (Takahashi et al., 2013), mutant TRBP expression plasmid or empty vector, 1 μg pOG 44 Flp-Recombinase Expression Vector (Life Technologies), and 6 μg polyethyleneimine (PEI, Sigma-Aldrich) were incubated at room temperature for 15 min and added to the medium. After 1 day, the culture medium was changed to the same medium containing 100 ng/ml Hygromycin B (Wako), and selected the cells expressing the wild-type or mutant TRBP for 1 month.

### Immunoprecipitation

One day before transfection, TRBP^−/−^ HeLa cells were inoculated at 6.0×10^5^ cells/well in a six-well cell culture plate. Cells were transfected with each TRBP expression plasmid with Lipofectamine 2000 (Invitrogen). Twenty-four hours after transfection, cells were washed with PBS and lysed in cold lysis Buffer [10 mM Hepes-NaOH (pH 7.9), 1.5 mM MgCl_2_, 10 mM KCl, 0.5 mM DTT, 140 mM NaCl, 1 mM EDTA, 1 mM Na_3_VO_4_, 10 mM NaF, 0.5% NP40, and 1×complete protease inhibitor]. RNasin Plus Ribonuclease Inhibitor (0.03 units/μl) was added to the lysis buffer to remove RNase. After centrifugation at 15,000 rpm for 10 min, the supernatant was further centrifuged at 48,000 rpm for 30 min. An aliquot of the recovered supernatant was used as an input sample. Immunoprecipitation was performed as previously reported (Takahashi et al., 2018).

In the experiment using Flp-In 293 TRBP-expressing cells, the cells were seeded at 6.0×10^5^ cells/well in a medium containing 1 μg/ml doxycycline (Dox) on a six-well cell culture plate. Two days later, the cells were collected with Trypsin-EDTA and washed with PBS. The immunoprecipitation with anti-FLAG antibody was carried out in the same manner for TRBP^−/−^ HeLa cells.

For purification of RNA interacting with TRBP, one day before transfection, Flp-In 293 cells expressing wild-type TRBP (TRBP-WT), mutant TRBP (TRBP-L326A/Y358A) and FLAG-tag expressing control (Empty) were cultured at 4.8×10^5^ cells/ml in 9-cm dishes in 10 ml medium containing 1 μg/ml Dox. Cells were transfected with pGL2-TAR-luciferase (5 µg) with PEI (15 µg). Four hours later, medium was exchanged to 10 ml of a fresh medium containing 1 μg/ml Dox. Immunoprecipitation was performed as described above.

### Western blot

After washing the cells (∼2×10^5^ cells) with PBS, 50 μl of RIPA Buffer [50 mM Tris-HCl (pH 8.0), 100 mM KCl, 0.5% NP 40, 10% Glycerol, 1 mM DTT, 1× complete protease inhibitor] was added and dissolved on ice for 20 min. After centrifugation at 7000×***g*** for 10 min, the supernatant was mixed with 2×SDS-PAGE Sample Buffer [4% SDS, 0.1 M Tris-HCl (pH 6.8), 12% 2-mercaptoethanol, 20% glycerol, 0.01% BPB] and transferred to polyvinylidene fluoride membrane using the TransBlot Turbo Transfer System (25 V, 30 min at 1 A) (BioRad). The membrane was blocked with 5% skim milk-TBST [20 mM Tris-HCl (pH 7.5), 150 mM NaCl, 0.2% Triton X-100] for 1 h, then incubated with a specific first antibody diluted in 5% skim milk-TBST overnight at 4°C. After washing the membrane three times with TBST, the membrane was incubated with the second antibody at room temperature for 1 h. After washing the membrane three times with TBST, the signal was detected with LAS 4000 mini (GE Healthcare) using ECL Prime Western Blotting Detection Reagents (GE Healthcare).

The first antibodies used were anti-FLAG antibody (1/1000, Cell Signaling Technology), anti-Dicer antibody (1/500) (Doi et al., 2003), anti-ribose polymerase (PARP) antibody (1/1000, abcam), anti-Caspase-3 antibody (1/400, abcam), anti-β-actin antibody (1/1000, Sigma-Aldrich). The second antibodies were Anti-rabbit IgG (1/5000, GE Healthcare) or Anti-mouse IgG (1/5000, GE Healthcare) labelled with HRP.

### Dual luciferase reporter assay to measure RNAi activity

One day before transfection, Flp-In 293 cells expressing wild-type TRBP (TRBP-WT), mutant TRBP (TRBP-L326A/Y358A) and FLAG-tag expressing control (Empty) were inoculated at a density of 10^5^ cells/well in a 24-well culture plate with a medium containing 1 μg/ml Dox. Cells were transfected with firefly *luciferase* expression plasmid (pGL3-control, Promega) (500 ng), *Renilla luciferase* expression plasmid (pRL-SV40, Promega) (100 ng) and shRNA expression plasmids (pSUPER-GY441 and pSUPER-FL774, Table S2) (0.5 µg) with PEI (1.5 µg). Four hours later, medium was exchanged to 1 ml of a fresh medium containing 1 μg/ml Dox. Twenty-four hours after transfection, the luminescence of firefly luciferase and *Renilla* luciferase was measured using Glomax-multi detection system (Promega).

### Preparation of ΔTAR luciferase expression plasmid

Using pGL2-TAR-luciferase as a template, PCR was performed with the primers shown in (Table S3) and KOD-Plus-Mutagenesis Kit (TOYOBO) to prepare a luciferase expression construct in which the TAR region was deleted (pGL2-ΔTAR-luciferase).

### Translational activity assay using luciferase reporter

One day before transfection, Flp-In 293 cells expressing wild-type TRBP (TRBP-WT), mutant TRBP (TRBP-L326A/Y358A) and FLAG-tag expressing control (Empty) were inoculated at a density of 10^5^ cells/well in a 24-well culture plate with a medium containing 1 μg/ml Dox. Cells were transfected with 1 µg of pGL2-TAR-luciferase and pRL-SV40 (100 ng) with PEI (3 µg). Four hours later, medium was exchanged to 1 ml of fresh medium containing 1 μg/ml Dox. At 24 h after transfection, the luminescence of firefly luciferase and that of *Renilla* luciferase were measured using Glomax-multi detection system (Promega).

### Real-time PCR

The same cells prepared for translational activity assays were used for measuring mRNA levels by real-time PCR. Total RNA was purified using RNeasy Mini Kit (Qiagen) and treated with DNaseI. cDNA was synthesized using High Capacity cDNA Reverse Transcription Kit (Applied Biosystems). Real time PCR was performed using Power SYBR Green PCR Master Mix (Applied Biosystems) by Step One Plus Real-Time PCR System (Applied Biosystems). The primer sequences used are shown in Table S4.

### Northern blot

Total RNA contained in the input sample and immunoprecipitated samples were extracted using ISOGEN (Nippon Gene), respectively, and then treated with RQ1 RNase-Free DNase (Promega) at 37°C for 30 min, and then RNA was extracted again using ISOGEN. Purified RNAs were loaded on a 10% denaturing polyacrylamide gel containing 8 M Urea (150 V, 60 min), and transferred to Hybond-N+ positive charged nylon membrane (GE Healthcare) (45 min at 25 V) and immobilized with CL-1000 Ultraviolet Crosslinker (UVP). The membrane was soaked in hybridization buffer (7% SDS, 5×SSC) At 37°C for 1 h to perform prehybridization. Hybridization was carried out overnight at 37°C using probe DNA (Table S5) labelled with γ-^32^P-ATP (PerkinElmer) at 5′-end for TAR RNA detection. The membrane was washed three times with Wash Buffer (2×SSC, 0.1% SDS) and detected with Typhoon FLA 9500 (GE Healthcare).

### Apoptosis assay

One day before transfection, TRBP knockout HeLa cells were inoculated at 2×10^6^ cells in a 9 cm dish. Cells were transfected with 3 μg of pGL2-TAR-luciferase or pGL2-ΔTAR-luciferase, along with pcDNA5-TRBP-WT, pcDNA5-TRBP-L326A/Y358A, or empty vector with Lipofectamine 2000 (Invitrogen). Four hours later, medium was exchanged into 10 ml of medium containing 10% FBS. Twenty-four hours after transfection, the cells were seeded at 2×10^5^ cells/well in a 12-well culture plate, and after 24 h, 20 ng of Tumor Necrosis Factor alpha (TNFα, Sigma-Aldrich) and 40 μg of cycloheximide (CHX, Wako) was added to the medium. At 6 and 10 h after TNFα/CHX treatment, the cells were photographed using Axiovert 200 microscope (ZEISS) and the cells were collected with Trypsin-EDTA. After washing the collected cells with PBS, 50 μl of RIPA Buffer was added and dissolved on ice for 20 min. After centrifugation at 7000×***g*** for 10 min, the supernatant was mixed with 2×SDS-PAGE Sample Buffer and used for western blotting. In addition, total RNA was also recovered and the amount of mRNAs of GIT2 and IER3 genes were quantified using specific primers for each gene (Table S4).

## Supplementary Material

Supplementary information
