# Correction: TRBP–Dicer interaction may enhance HIV-1 TAR RNA translation via TAR RNA processing, repressing host-cell apoptosis

**DOI:** 10.1242/bio.059748

**Published:** 2022-12-23

**Authors:** Chiaki Komori, Tomoko Takahashi, Yuko Nakano, Kumiko Ui-Tei

There was an error published in *Biol. Open* (2020) **9**, bio050435 (doi:10.1242/bio.050435)

In Fig. 5A, an incorrect image was used for the 10 h –TNFα/CHX TRBP-WT panel. The corrected and original panels are shown below; the online version of the article and pdf have been updated.

**Figure:**
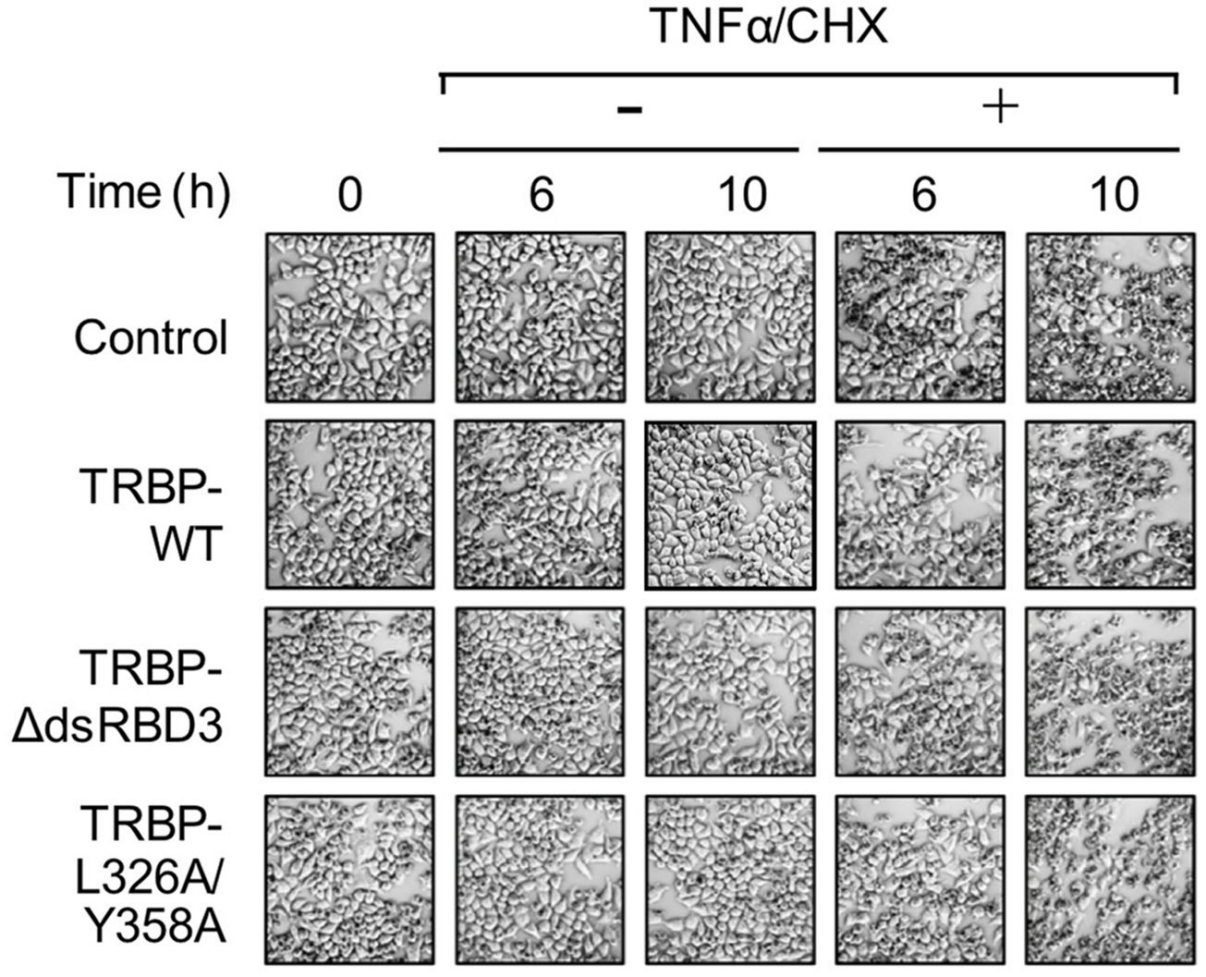
**Fig. 5A (corrected). Regulation of apoptosis by the TRBP–Dicer interaction.** (A) Morphological changes in TRBP^−/−^ HeLa cells transfected with pGL2-TAR-Luciferase along with each expression plasmid of control FLAG-tag alone, FLAG-tagged TRBP-WT, TRBPdsRBDΔ3 or TRBP-L326A/Y358A at 0, 6 and 10 h after treatment with or without TNFα/CHX.

The authors apologise to readers for the error, which does not impact the results or conclusions of this paper.

**Figure:**
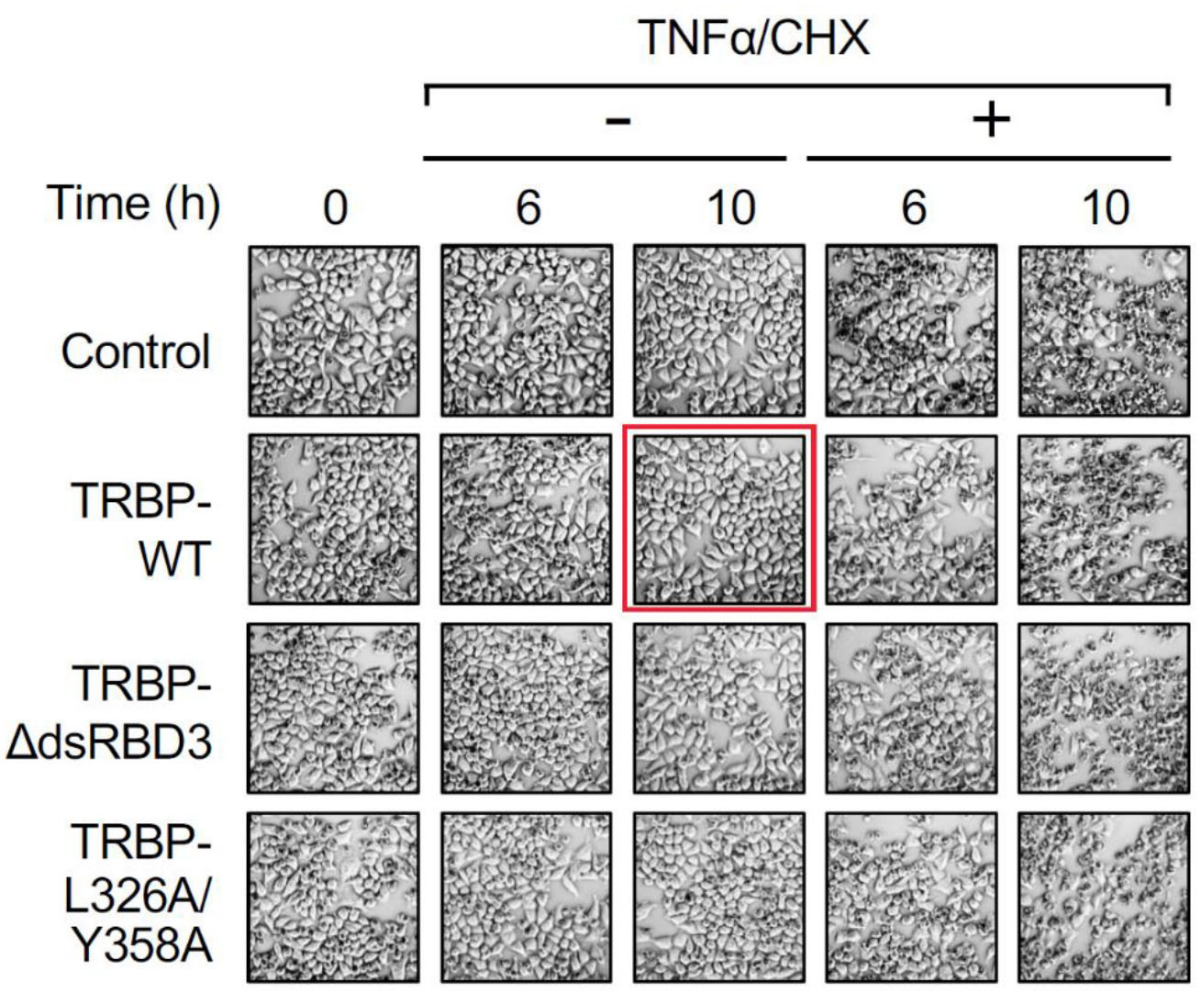
**Fig. 5A (original). Regulation of apoptosis by the TRBP–Dicer interaction.** (A) Morphological changes in TRBP^−/−^ HeLa cells transfected with pGL2-TAR-Luciferase along with each expression plasmid of control FLAG-tag alone, FLAG-tagged TRBP-WT, TRBPdsRBDΔ3 or TRBP-L326A/Y358A at 0, 6 and 10 h after treatment with or without TNFα/CHX.

