# Supplementary material for: TRBP–Dicer interaction may enhance HIV-1 TAR RNA translation via TAR RNA processing, repressing host-cell apoptosis
Source: Biol Open. 2020 Feb 25;9(2):bio050435. doi: 10.1242/bio.050435 (PMC7055394; doi:10.1242/bio.050435)
Supplement: Supplementary information [file biolopen-9-050435-s1.pdf]

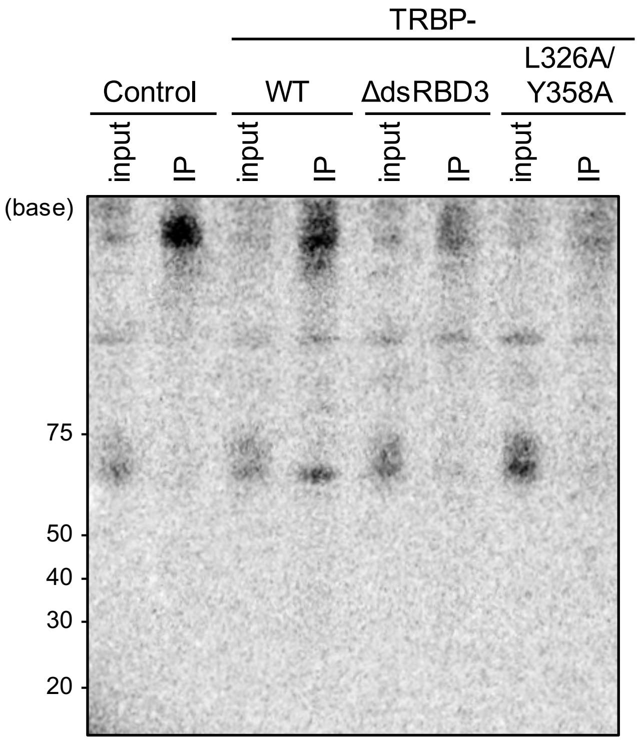

Fig. S1. Northern blot analysis of the excision of TAR miRNA. Flp-In 293 cells with control FLAG-tag alone, FLAG-tagged TRBP-WT, TRBP-dsRBDΔ3, or TRBP-L326A/Y358A were transfected with pGL2-TAR-Luciferase, and IP was performed with anti-FLAG antibody. Northern blotting was performed using purified total RNA from input samples and IP samples with a probe for detecting the 5′ stem region of TAR RNA (red). A band of approximately 60 bases corresponding to the cleaved stem-loop-structured TAR RNA was observed in the IP sample of TRBP-WT, but not in those of TRBP-dsRBDΔ3 or TRBPL326A/Y358A.

Table S1

PCR primers used for generating mutant TRBP expression plasmids.

Primer name	Sequence (5' to 3')
L326A-F	cgcagtggaactgtccacccagccggcca
L326A-R	cactggcagagtccactcaggctcag
V336A_H338A-F	gtgcaggctctgcaaccaccagggaggcag
V336A_H338A-R	atgcagtggccggctgggtggacagttccac
Y358A-F	ctcaagatcatggcaggcagcaagtg
Y358A-R	tgcctgcagggcacggcgggcagcctc

F indicates forward primer. R, reverse primer.

Table S2

siRNA sequences expected to be cleaved out from shRNAs used for dual luciferase reporter assay.

shRNA	Guide strand sequence (5' to 3')	Passenger strand sequence (5' to 3')
pSUPER GY441	gccacaacgucuauaucaugg	augauauagacguuguggcug
pSUPER FL774	auuaagacgacucgaaaucca	gauuucgagucgucuuaaugu

Biology Open • Supplementary information

Table S3

PCR primers used for generating pGL2-ΔTAR-luciferase plasmid.

Primer name	Sequence (5' to 3')
3ΔTAR-F	actgcttaagcctcaataaagcttgg
3ΔTAR-R	agtacaggcaaaaagcagctgcttat

F indicates forward primer. R, reverse primer.

Biology Open • Supplementary information

Table S4

PCR primers used for qRT-PCR.

Primer name	Sequence (5' to 3')
hGAPDH-F	tgcaccaccaactgcttag
hGAPDH-R	agaggcagggatgatgttc
pGL2-luc-F	gaaccgctggagagcaactg
pGL2-luc-R	tagcttctgccaaccgaacg
hTubulinB-F	ctggcaccatggactctg
hTubulinB-R	tcggctccctctgtgtag
GIT2-F	aacaccccactccatgttgc
GIT2-R	tgccctccttgccttgcataa
IER3-F	acttcggagccctcggacta
IER3-R	gagtgcggggagtcacagtt

F indicates forward primer. R, reverse primer.

Table S5

DNA probe used for northern blot.

Probe name	Sequence (5' to 3')
TAR_RNA	gggttccctagttagccagagagc

Biology Open • Supplementary information
